# Low bone mineral density in Thai children with systemic lupus erythematosus: prevalence and risk factors

**DOI:** 10.2478/abm-2021-0030

**Published:** 2021-10-29

**Authors:** Ankanee Chanakul, Suriyaphon Khunrattanaphon, Tawatchai Deekajorndech

**Affiliations:** Division of Nephrology, Department of Pediatrics, Faculty of Medicine, Chulalongkorn University, Bangkok 10330, Thailand; Department of Pediatrics, Faculty of Medicine, Chulalongkorn University, Bangkok 10330, Thailand

**Keywords:** juvenile-onset, lupus erythematosus, systemic, osteoporosis, steroids, Thai

## Abstract

**Background:**

Improvement of disease recognition and management has increased the survival of children with systemic lupus erythematosus (SLE), but has shifted the morbidity focus toward long-term complications, such as low bone mass and osteoporosis. Studies in adults with SLE show older age, chronic inflammation, and corticosteroid therapy are risk factors for low bone mineral density (BMD) and osteoporosis.

**Objectives:**

To determine the prevalence of and identify risk factors associated with low BMD in Thai children with SLE.

**Methods:**

We conducted a retrospective review of demographic data and clinical variables for a cohort of 60 Thai children with SLE who underwent 2 dual-energy X-ray absorptiometry (DXA) at their initial examination and later follow-up. We considered a BMD *z* score ≤ −2.0 to indicate low BMD. Binary logistic regression was used to assess risk factors potentially associated with low BMD.

**Results:**

The prevalence of low BMD at the first visit was 40% and increased to 55% over follow-up. We found a significantly decreased hip BMD *z* score (median difference −0.25, 95% confidence interval [CI] −0.40 to −0.05; *P* = 0.016) and lumbar BMD *z* score (median difference −0.49, 95% CI −0.69 to −0.28; *P* < 0.001) over time. The cumulative steroid dose tended to be higher for patients with low BMD (adjusted odds ratio [OR] = 1.08, 95% CI 1.00 to 1.17; *P* = 0.050).

**Conclusion:**

Low BMD has a 40% prevalence in Thai children newly diagnosed with SLE and progresses significantly over time. Higher cumulative corticosteroid dose tended to be associated with a low BMD, but we did not find a significant risk in this small sample.

Systemic lupus erythematosus (SLE) is a chronic inflammatory autoimmune disease that primarily affects women of reproductive age. However, around 20% of cases present during childhood and adolescence [1, 2]. Juvenile-onset SLE not only differs from adult-onset SLE in clinical manifestations and serological characteristics, but also tends to involve major organs more frequently than the adult-onset disease and include more serious clinical outcomes [2, 3]. Due to the more aggressive clinical features of the disease in juvenile-onset SLE, children tend to be given more intensive drug therapy including prolonged courses of corticosteroid therapy. The life expectancy of children with SLE has recently improved. However, now they face a range of comorbidities resulting from sequelae of disease and medication side effects. Low bone mineral density (BMD) and osteoporosis contribute to major health problems in adults as well as in children with SLE [4,5,6,7,8,9].

Osteoporosis is defined as “a skeletal disease characterised by low bone mass and microarchitectural deterioration with a resulting increase in bone fragility and hence susceptibility to fracture” [10]. Bone mass increases throughout childhood and adolescence and achieves a peak by the age of 20 years [11]. Patients with juvenile-onset SLE are at risk of low bone mass in adulthood because the development of maximal bone mass is lower than expected during the period of skeletal growth and without signs of catch-up of BMD later in adulthood. A variety of potential risks for low bone mass exist in patients with SLE. The inflammatory responses to autoimmune diseases alter bone remodeling and reduce BMD [12]. Corticosteroids impair the microarchitecture of bone tissue and result in a decline of bone strength [13, 14]. Corticosteroids also have an influence on sex hormone production, decrease calcium absorption from the intestines, and increase urinary calcium excretion, which contributes to bone fragility. Other potential risks such as vitamin D deficiency caused by limited sun exposure, nutritional deficiency, or reduced physical activity may negatively affect bone health in patients with SLE.

Dual-energy X-ray absorptiometry (DXA) is the most widely used technique for measuring areal BMD in children and adolescents. The lumbar spine (L1–L4) and total body, but not including the head, are the common skeletal sites suitable for measuring the areal BMD [15]. The BMD test results are used to calculate a *z* score (an estimate of the standard deviations (SDs) from the expected mean) based on the mean reference values for healthy children of the same age, sex, and ancestry [16]. The International Society of Clinical Densitometry’s (ISCD’s) updated consensus in 2013 has defined low BMD for the pediatric DXA report as BMD *z* scores ≤ −2.0 SD [17]. Although low BMD is a strong predictor of fracture in the elderly, evidence for the association between low BMD and risk of fracture in children is limited [18, 19].

DXA has an important role in monitoring bone health in children and adolescents with SLE. There are few studies that have evaluated bone health in children with SLE and results have varied greatly due to the various definitions. We conducted a retrospective review of medical records of children with SLE who had a BMD measurement to determine the prevalence of low BMD and investigated the factors potentially associated with a risk of low BMD in these patients.

## Methods

### Patients

We conducted a retrospective review of medical records of patients who had onset of SLE before the age of 18 years, fulfilling at least 4 of the American College Rheumatology diagnostic criteria [20]. All patients attended the Pediatric Nephrology Clinic between 2010 and 2015 at the King Chulalongkorn Memorial Hospital, a large 1435 bed general and tertiary referral teaching hospital in Bangkok. Patients with previously diagnosed chronic kidney disease based on an estimated glomerular filtration rate declined to <60 mL/min/1.73 m^2^ for 3 months were excluded from the study. Participants underwent DXA measurements at the hip and lumbar spine BMD and repeated DXA during follow-up as a part of their routine care in the clinic. Demographic and clinical characteristics including sex, age, weight, height, disease duration before DXA scan, history of fracture, and use of medications were recorded. The SLE Disease Activity Index (SLEDAI) score and serum level of complement component 3 (C3) were used to assess SLE disease activity. Prescribed medications including glucocorticoid, immunosuppressants, calcium, and vitamin D supplements were recorded. Pubertal status and physical activity could not be determined by this retrospective chart review. The study was approved by the Institutional Research Ethics Review Board, Faculty of Medicine, Chulalongkorn University (IRB No. 149/59) and complies with the principles outlined in the contemporary revision of the Declaration of Helsinki 1964 (World Medical Association) incorporating the most recent (2013) and earlier amendments, and international guidelines for human research protection detailed in the Belmont Report, Council for International Organizations of Medical Services Guidelines, and the International Conference on Harmonization in Good Clinical Practice. We used the cohort guidelines in the STROBE statement to ensure complete reporting of this observational study [21].

### BMD measurement

DXA had been performed to measure BMD of the lumbar spine (L1–L4) and hip as recommended by the American College of Radiology as suitable for pediatric patients [22]. BMD measurement is reported as a *z* score calculated based on age and sex-matched reference values. Low BMD was defined by a BMD *z* score ≤ −2 [15,16,17].

### Statistical analysis

Demographic data is summarized using descriptive statistics and expressed as mean with SD for variables with a normal distribution, as median and interquartile ranges (IQRs) for those without normal distribution, or as a percentage for categorical variables. Data between groups were compared using either an independent *t* test, Mann–Whitney *U* test, or χ^2^ test, as appropriate. A Wilcoxon signed-rank test was used to determine differences in mean values from the initial and the follow-up DXAs. Factors associated with low BMD were analyzed using univariate logistic regression. Multivariate logistic regression was performed for variables with *P* < 0.1 from univariate analysis and are expressed as an adjusted odds ratio (OR). These statistical analyses were performed using IBM Statistics for Windows (version 21). Power analysis was based on the BMD of the lumbar spine at baseline and at the last follow-up using Stata (version 14, StataCorp). For all statistical analyses, *P* < 0.05 in a two-tailed test was considered significant.

## Results

### Patient characteristics

We considered the demographic and clinical data from medical records of a cohort of 60 pediatric patients with SLE in this retrospective observational study. The majority of the patients (52, 87%) were girls, yielding a female-to-male ratio of 6.5:1. The mean age of all patients at diagnosis was 11 ± 2.5 years. The disease duration from the diagnosis to the first DXA ranged from 2 months to 58 months and to follow-up BMD measurement ranged from 11 months to 87 months, respectively. The most common renal pathology was proliferative nephritis (52%). All patients had been receiving steroid treatment at the time of the first DXA with a cumulative steroid dose ranging from 1.9 g to 42.6 g. None of our patients had a history of fracture. Demographic information including disease activity, medications, and laboratory results are summarized in **Table 1**.

**Table 1:** Characteristics based on the BMD z score in pediatric patients with SLE

**Characteristic**	**First DXA (n = 60)**			**Follow-up DXA (n = 51)**		

	**BMD *z* score**		***P***	**BMD *z* score**		***P***
	
	**> −2 (n = 36)**	**≤−2 (n = 24)**		**>−2 (n = 23)**	**≤−2 (n = 28)**	
Age at time of DXA (years), mean (SD)	11.34 (2.42)	11.32 (2.72)	0.98†	10.62 (2.67)	11.38 (2.05)	0.28†
Disease duration (months), median (IQR)	13.5 (7–21)	17 (8.5–32.5)	0.39‡	26 (22–37)	40.5 (20.5–53.5)	0.31‡
Weight for age *z* score, mean (SD)	1.05 (1.64)	–0.12 (1.78)	0.014†^*^	0.92 (1.98)	0.16 (1.56)	0.15†
Height for age *z* score, mean (SD)	–0.58 (1.46)	–1.00 (1.01)	0.24†	–0.58 (1.07)	–0.92 (1.19)	0.32†
SLEDAI score at DXA, mean (SD)	5.21 (5.73)	6.29 (4.94)	0.48†	4.25 (2.90)	5.89 (4.24)	0.25†
C3 level, mean (SD)	89.31 (29.30)	88.11 (36.16)	0.89†	93.40 (21.10)	99.31 (28.10)	0.40†
Vitamin D level, median (IQR)	20 (8–29)	30.50 (13.5–60)	0.26‡	24.5 (19.6–33.1)	20.1 (13.3–29.2)	0.41‡
Cumulative corticosteroid dose (g), mean (SD)	11.70 (7.00)	17.38 (13.58)	0.10†	15.91 (6.19)	23.65 (14.62)	0.059†
Immunosuppressant, n (%)	25 (69.4)	17 (70.8)	0.53§	18 (78.3)	23 (82.1)	0.73§
Vitamin D supplement, n (%)	17 (47.2)	10 (41.7)	0.53§	13 (56.5)	20 (71.4)	0.61§
Calcium supplement, n (%)	19 (52.8)	7 (29.2)	0.16§	18 (78.3)	17 (60.7)	0.64§

BMD, bone mineral density; DXA, dual-energy X-ray absorptiometry; IQR, interquartile range; SD, standard deviation; SLE, systemic lupus erythematosus; SLEDAI, SLE disease activity index.

† Independent *t* test;

‡ Mann–Whitney *U* test;

§ χ^2^ test;

* *P* < 0.05.

### BMD at the hip and lumbar spine

**Figure 1:**
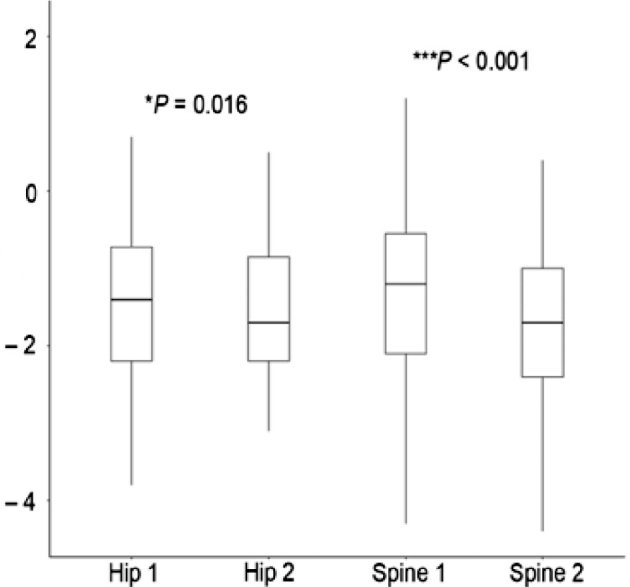
The difference in BMD *z* scores between the first and the follow-up DXA of the hip and lumbar spine. Hip 1, first DXA scan of the hip; Hip 2, follow-up DXA scan of the hip; LS 1, first DXA scan of the lumbar spine; LS 2, follow-up DXA scan of the lumbar spine. Wilcoxon signed-rank test; *P < 0.05, ***P < 0.001. BMD, bone mineral density; DXA, dual-energy X-ray absorptiometry.

Of the 60 patients who had a DXA scan performed for the first time, 24 had a BMD *z* score ≤ −2, demonstrating a prevalence of low BMD of 40%. Only 51 patients had a follow-up DXA scan completed. At follow-up, 28 patients had a BMD *z* score ≤ −2, which indicates an increased prevalence of low BMD to 55%. Comparison between the first and the follow-up DXA showed a significant decrease in both hip BMD *z* score (median difference −0.25, 95% confidence interval [CI] −0.40 to −0.05; *P* = 0.016) and lumbar spine BMD *z* score (median difference: −0.49, 95% CI −0.69 to −0.28; *P* < 0.001) (**Figure 1**).

### Factors associated with low BMD

There was no significant association between low BMD and age at the time of BMD measurement, disease duration, disease activity, vitamin D level, or the intake of either immunosuppressant drugs or vitamin D supplements. Mean weight for age *z* score in patients with low BMD was significantly lower than in patients with a BMD *z* score > −2 and was significant in univariate but not in multivariate analyses. The cumulative dose of steroids in patients with BMD score ≤ −2 tended to be higher than patients with a BMD *z* score > −2 (adjusted OR = 1.08, 95% CI 1.0 to 1.17; *P* = 0.050); however, this tendency was not significant **(Table 2**).

**Table 2:** Factors associated with low BMD in patients with childhood SLE

**Characteristic**	**First DXA**				**Follow-up DXA**			
	
	**Univariate analysis**		**Multivariate analysis**		**Univariate analysis**		**Multivariate analysis**	
	**OR (95% CI)**	***P* †**	**Adjusted OR (95% CI)**	***P* ¶**	**OR (95% CI)**	***P* †**	**Adjusted OR (95% CI)**	***P* ¶**
Weight for age *z* score	0.67 (0.45–0.93)	0.03	0.78 (0.50–1.17)	0.25	0.87 (0.62–1.20)	0.394		
Cumulative steroid dose	1.06 (0.99–1.13)	0.10	1.00 (1.00–1.00)	0.10	1.07 (0.99–1.16)	0.059	1.08 (1.00–1.17)	0.050

Weight for age *z* score and cumulative steroid dose were included in the forward stepwise model of multiple logistic regression.

BMD, bone mineral density; CI, confidence interval; DXA, dual-energy X-ray absorptiometry, OR, odds ratio; SLE, systemic lupus erythematosus.

† Independent *t* test;

¶ stepwise multiple logistic regression.

## Discussion

Children with SLE are susceptible to bone loss, osteoporosis, and fragility fracture due to disease development before reaching their peak bone mass. The high frequency of osteopenia in juvenile-onset SLE has been addressed in a few studies [7,8,9, 23]. Bone fractures are more likely to occur in SLE patients. A high frequency of vertebral fractures of up to 23% has been reported for patients with juvenile-onset SLE [24]. We defined low BMD for age as a *z* score −2 SD and found a high prevalence of low BMD in children with SLE of 40% with an increasing prevalence of 55% over follow-up. No patients whose data were included in the present study had a previous fragility fracture. The significant decrease in BMD *z* score at the lumbar spine found during follow-up was consistent with other studies, which indicated corticosteroids had an effect on the metabolism of bone, leading to bone loss, which occurs preferentially in trabecular bone, like lumbar vertebrae [8, 25, 26]. Some studies have found the cumulative corticosteroid dose was significantly associated with reduced BMD [7,8,9], while Castro et al. found no significant correlation [27]. The present study suggested a tendency for the cumulative dose of steroids to be higher in patients with low BMD; however, the tendency was not significant.

Optimal intake of calcium and vitamin D is crucial for the healthy mineralization of the skeleton. Vitamin D insufficiency and low levels of calcium contribute to reduced BMD and the appearance of fractures. Several factors lead to a greater risk of developing low levels of plasma vitamin D in patients with SLE, including low vitamin D intake, avoidance of sunshine, and the inflammatory process of the disease itself. However, we did not find a significant association between low BMD and vitamin D deficiency. There is a trend toward a lower risk of osteoporosis and fracture in patients who were treated with calcium and vitamin D, but the association was not significant. Despite unproven efficacy, calcium and vitamin D supplementation are recommended for all patients who undergo long-term use of steroids. The apparent lack of a significant association between low BMD and the following factors, such as disease activity, disease duration, and immunosuppressive drug use, may be attributed to the small sample size of patients whose data was included in the present study. This limitation corresponded to the low statistical power (calculated at 0.37) for assessing the relationship between any individual factor and low BMD. Furthermore, the retrospective nature of the present study limited the data available, which was unable to reflect all changes of risk factors over time. A longitudinal study with a greater sample size should be considered to clarify the association between potentially important risk factors and low BMD in Thai children with SLE.

## Conclusions

We found a low BMD prevalence of approximately 40% in Thai children newly diagnosed with SLE. This prevalence increased and significant reductions in hip and lumbar BMD were found over time. This lower BMD may lead to a greater risk of developing osteoporosis and fragility fractures. Patients with a higher cumulative corticosteroid dose tended to have a low BMD, but the risk was not significant. We strongly recommend BMD DXA for initial screening and be repeated annually to assess the risk of osteoporosis and fragility fractures, and to improve their management. A longitudinal study with a larger sample size should be considered to clarify the association between potential risk factors such as cumulative corticosteroid dose, vitamin D deficiency caused by limited sun exposure, nutritional deficiency, or reduced physical activity, and low BMD in Thai children with SLE.

## References

[1] Rosenberg AM (1994). Systemic lupus erythematosus in children. Springer Semin Immunopathol.

[2] Tarr T, Dérfalvi B, Győri N, Szántó A, Siminszky Z, Malik A (2015). Similarities and differences between pediatric and adult patients with systemic lupus erythematosus. Lupus.

[3] Amaral B, Murphy G, Ioannou Y, Isenberg DA (2014). A comparison of the outcome of adolescent and adult-onset systemic lupus erythematosus. Rheumatology (Oxford).

[4] Kipen Y, Buchbinder R, Forbes A, Strauss B, Littlejohn G, Morand E (1997). Prevalence of reduced bone mineral density in systemic lupus erythematosus and the role of steroids. J Rheumatol.

[5] Lakshminarayanan S, Walsh S, Mohanraj M, Rothfield N (2001). Factors associated with low bone mineral density in female patients with systemic lupus erythematosus. J Rheumatol.

[6] Bultink IEM, Lems WF, Kostense PJ, Dijkmans BAC, Voskuyl AE (2005). Prevalence of and risk factors for low bone mineral density and vertebral fractures in patients with systemic lupus erythematosus. Arthritis Rheum.

[7] Trapani S, Civinini R, Ermini M, Paci E, Falcini F (1998). Osteoporosis in juvenile systemic lupus erythematosus: a longitudinal study on the effect of steroids on bone mineral density. Rheumatol Int.

[8] Lilleby V, Lien G, Frøslie KF, Haugen M, Flatø B, Førre Ø (2005). Frequency of osteopenia in children and young adults with childhood-onset systemic lupus erythematosus. Arthritis Rheum.

[9] Compeyrot-Lacassagne S, Tyrrell PN, Atenafu E, Doria AS, Stephens D, Gilday D, Silverman ED (2007). Prevalence and etiology of low bone mineral density in juvenile systemic lupus erythematosus. Arthritis Rheum.

[10] Sambrook P, Cooper C (2006). Osteoporosis. Lancet.

[11] Boot AM, de Ridder MAJ, van der Sluis IM, van Slobbe I, Krenning EP, Keizer-Schrama SMPF (2010). Peak bone mineral density, lean body mass and fractures. Bone.

[12] Lane NE (2006). Therapy insight: osteoporosis and osteonecrosis in systemic lupus erythematosus. Nat Clin Pract Rheumatol.

[13] Lilleby V (2007). Bone status in juvenile systemic lupus erythematosus. Lupus.

[14] Sarinho ESC, Melo VMPP (2017). Glucocorticoid-induced bone disease: mechanism and importance in pediatric practice. Rev Paul Pediatr.

[15] Crabtree NJ, Arabi A, Bachrach LK, Fewtrell M, El-Hajj Fuleihan G, Kecskemethy HH (2014). Dual-energy X-ray absorptiometry interpretation and reporting in children and adolescents: the revised 2013 ISCD Pediatric Official Positions. J Clin Densitom.

[16] Bachrach LK, Gordon CM (2016). Section on Endocrinology. Bone densitometry in children and adolescents. Pediatrics.

[17] Gordon CM, Leonard MB, Zemel BS, International Society for Clinical Densitometry (2014). 2013 Pediatric Position Development Conference: executive summary and reflections. J Clin Densitom.

[18] Kanis JA, Oden A, Johnell O, Johansson H, De Laet C, Brown J (2007). The use of clinical risk factors enhances the performance of BMD in the prediction of hip and osteoporotic fractures in men and women. Osteoporos Int.

[19] Bishop N, Arundel P, Clark E, Dimitri P, Farr J, Jones G (2014). Fracture prediction and the definition of osteoporosis in children and adolescents: the ISCD 2013 Pediatric Official Positions. J Clin Densitom.

[20] Hochberg MC (1997). Updating the American College of Rheumatology revised criteria for the classification of systemic lupus erythematosus. Arthritis Rheum.

[21] von Elm E, Altman DG, Egger M, Pocock SJ, Gotzsche PC, Vandenbroucke JP (2007). The Strengthening the Reporting of Observational Studies in Epidemiology (STROBE) Statement: guidelines for reporting observational studies. Ann Intern Med.

[22] Binkovitz LA, Henwood MJ (2007). Pediatric DXA: technique and interpretation. Pediatr Radiol.

[23] Alsufyani KA, Ortiz-Alvarez O, Cabral DA, Tucker LB, Petty RE, Nadel H, Malleson PM (2005). Bone mineral density in children and adolescents with systemic lupus erythematosus, juvenile dermatomyositis, and systemic vasculitis: relationship to disease duration, cumulative corticosteroid dose, calcium intake, and exercise. J Rheumatol.

[24] Regio PL, Bonfá E, Takayama L, Pereira RMR (2008). The influence of lean mass in trabecular and cortical bone in juvenile onset systemic lupus erythematosus. Lupus.

[25] van Staa TP, Leufkens HG, Cooper C (2002). The epidemiology of corticosteroid-induced osteoporosis: a meta-analysis. Osteoporosis Int.

[26] De Nijs RNJ (2008). Glucocorticoid-induced osteoporosis: a review on pathophysiology and treatment options. Minerva Med.

[27] Castro TC, Terreri MT, Szejnfeld VL, Castro CH, Fisberg M, Gabay M, Hilário MOE (2002). Bone mineral density in juvenile systemic lupus nephritis. Braz J Med Biol Res.

